# Platelet Indices as Unequivocal Markers of Active Disease in Patients With Nonradiographic Axial Spondyloarthritis: Protocol for a Cross-Sectional Study

**DOI:** 10.2196/71909

**Published:** 2025-11-24

**Authors:** Abhijeet Kumar Agrawal, Sourya Acharya, Jahnabi Bhagawati, Shivali Kashikar, Nishant Raj

**Affiliations:** 1 Datta Meghe Institute of Higher Education and Research Wardha, Maharashtra India

**Keywords:** nonradiographic axial spondyloarthritis, ankylosing spondylitis, platelet indices, sacroiliitis, CRP-negative spondyloarthritis, C-reactive protein

## Abstract

**Background:**

Spondyloarthritis is divided into axial and peripheral subtypes. The axial subtype is further divided into ankylosing spondylitis (AS) or radiographic axial spondyloarthritis and nonradiographic axial spondyloarthritis (nr-axSpA). Although AS and nr-axSpA share some common features, nr-axSpA lacks X-ray–based sacroiliitis as defined by the modified New York criteria, that is, the presence of either bilateral grade 2 sacroiliitis or unilateral grade 3 sacroiliitis, which is present in AS. Disease activity in either of them is measured via C-reactive protein (CRP) levels or magnetic resonance imaging (MRI) of the pelvis showing sacroiliitis due to the presence of bone marrow edema. A person with active disease can have normal CRP and MRI findings, especially in nr-axSpA. Immune system activation during inflammation has been shown to alter platelet maturation and morphology, as reflected by platelet indices. These platelet indices have been studied in the past in various autoimmune diseases, such as psoriatic arthritis, AS, and rheumatoid arthritis, and have been correlated with disease activity. Particularly, in spondyloarthritis, platelet indices are more central to the pathology of sacroiliitis, and CRP, which is currently used, is a generalized marker of inflammation. Therefore, platelet indices can provide a better understanding of inflammation, particularly in patients with sacroiliitis.

**Objective:**

This study aims to investigate whether platelet indices can be better markers of inflammation in patients with nr-axSpA. We will determine whether platelet indices are reliable biomarkers for measuring disease activity in patients with nr-axSpA.

**Methods:**

All patients who are classified as having axial spondyloarthritis as per the Assessment of Spondylarthritis International Society criteria will be included in this study. Patients will be divided into 2 case groups: group A (patients without radiographic sacroiliitis [nr-axSpA]) and group B (patients with radiographic sacroiliitis [AS]). Healthy individuals will be enrolled as controls to compare and correlate platelet indices such as platelet count, plateletcrit, mean platelet volume, and platelet distribution width with CRP, erythrocyte sedimentation rate, and MRI findings in patients with AS and nr-axSpA.

**Results:**

This is a nonfunded study. The data collection started on February 1, 2025, and we expect to complete the study by December 2027. As of July 2025, 7 individuals had been enrolled in groups A and B, respectively, and 5 individuals had been included in the control group. At the end of the study, we will be able to correlate whether platelet indices are trustworthy biomarkers of active disease in patients with nr-axSpA.

**Conclusions:**

If platelet indices prove to be better markers of active disease in patients with nr-AxSpA, then these markers should be included in the routine evaluation of these patients.

**International Registered Report Identifier (IRRID):**

DERR1-10.2196/71909

## Introduction

### Background

Spondyloarthritis is divided into axial and peripheral subtypes. The axial subtype is further subdivided into ankylosing spondylitis (AS) or radiographic axial spondyloarthritis (r-axSpA) and nonradiographic axial spondyloarthritis (nr-axSpA). Both represent conditions with inflammatory back pain that start in individuals aged 45 years or younger and are present for at least 3 months, as per the Assessment of Spondyloarthritis International Society (ASAS) criteria. Although both AS and nr-axSpA share some common features, nr-axSpA lacks X-ray–based sacroiliitis as defined by the modified New York criteria [1,2].

Patients with AS were diagnosed on the basis of the modified New York criteria, which had a sensitivity of 95% and specificity of 85% [3]. However, this criterion led to delayed diagnosis of patients in the early stages of inflammation as no magnetic resonance imaging (MRI) findings were included to identify the active inflammation of sacroiliac joints that were not visible on conventional X-rays, and neither did it include a separate criterion for nonradiological spondyloarthritis, a subgroup of patients who may lack any radiological findings of inflammation of the sacroiliac region. With the advent of newer imaging modalities, such as computed tomography scans and MRIs, newer criteria, such as the Amor criteria [4] and the European criteria [5], were developed for early diagnosis of inflammation of the axial skeleton. Subsequently, the ASAS criteria [6] provided a new classification criterion that included both AS and nr-axSpA. This is currently applied as a classification criterion and has a sensitivity and specificity of 66.2% and 97.5%, respectively, for the imaging arm and 80% for the clinical arm. Although this is a classification criterion and should not be used for diagnostic purposes, it is the only criterion encompassing both early and late phenotypes of spondyloarthritis [6].

Labeling of the disease as active often requires either raised C-reactive protein (CRP) levels or an MRI showing active lesions in the sacroiliac joint, along with a clinical score showing disease activity [7]. A high CRP level or active lesions on MRI facilitates the use of anti–tumor necrosis factor (TNF) therapy for patients. CRP levels also predict the response to treatment with biologicals (anti-TNF). Higher CRP levels during the initial evaluation indicate a better response to anti-TNF agents [7]. Similar to patients with AS, patients with nr-axSpA also need to fulfill these criteria for the use of anti-TNF agents [8]. Apart from anti-TNF agents, other drugs that are used are interleukin 17 inhibitors and Janus kinase inhibitors such as tofacitinib, sulfasalazine, and etoricoxib [9].

The literature shows that patients with clinically active disease can have normal CRP levels and MRI findings. Unfortunately, patients with active disease without evidence of inflammation are not eligible for anti-TNF therapy and are considered CRP-negative. As there is no objective evidence of inflammation, such patients present a dilemma for rheumatologists when deciding whether to escalate therapy [7]. MRI, too, may not show active inflammation in patients with active disease if bone marrow edema is below a certain threshold, and such lesions can be detected only via bone biopsy, which is invasive [8].

Such patients need alternative and reliable markers of active inflammation, as early escalation of therapy can control their symptoms and prevent or slow down radiographic progression.

The 2 entities, r-axSpA and nr-axSpA, were devised to include the entire spectrum of axial spondyloarthritis and its staging for research purposes. There is some controversy regarding whether to consider them as the same disease. Nr-axSpA is distinct in other aspects, such as a shorter duration of disease, self-limiting nature, and lower CRP levels; however, the actual clinical burden may remain at par with r-axSpA [10].

Hence, it is imperative that patients with nr-axSpA, who are more likely to be CRP-negative or normal and may not show sacroiliac joint lesions on MRI, should be assessed for alternate markers of active disease [11]. This emphasizes the need for more biomarkers in patients with nr-axSpA, as they are more prone to false-negative CRP or MRI results.

Various studies have shown that platelet indices used in numerous rheumatological diseases, such as psoriasis, rheumatoid arthritis, AS, and scleroderma, correlate well with inflammatory markers and clinical scores of disease activity [12-15]. However, no study, in particular, has been conducted in patients with nr-axSpA, where the probability of having a normal CRP or MRI is much higher.

Activation of the immune system causes the release of inflammatory cytokines, autoantibodies, and immune complexes. When chronic, inflammation may influence thrombopoiesis and erythropoiesis, thereby altering the properties of different blood indices such as red cell distribution width (RDW), platelet distribution width (PDW), and mean platelet volume (MPV). There may be changes in platelet maturation and morphology. Parameters such as MPV have shown an inverse correlation with acute-phase reactants and joint involvement in spondyloarthropathies [16].

Byun et al [17] showed PDW correlated well with disease activity measures such as the Bath Ankylosing Spondylitis Disease Activity Index (BASDAI) and Ankylosing Spondylitis Disease Activity Score (ASDAS)-CRP in patients with AS, even in patients whose traditional inflammatory markers were within the normal range. Platelet count (PC) also correlated with CRP levels [14]. However, this study did not include platelet indices such as plateletcrit and MPV. Moreover, no comparison was made with a control group. The study did not include any other biomarkers apart from platelet indices. The study also had no separate analysis for the nr-axSpA, which has a greater propensity of not showing any objective signs of inflammation or disease activity.

A retrospective study showed that plateletcrit was a positive marker of active inflammation, whereas MPV and PDW were negative markers in patients with rheumatoid arthritis [13]. Again, this study did not include PC among the indices.

Targońska-Stępniak et al [18] showed that in rheumatoid arthritis, PC and plateletcrit proved to be reliable positive markers of inflammation, and PDW was a negative marker. The study showed that these were inexpensive and reliable markers that correlated well with traditional inflammatory markers such as erythrocyte sedimentation rate (ESR) and CRP, clinical scores such as disease activity score and ultrasound findings and scoring (power Doppler ultrasonography) [18]. The study omitted MPV in its analysis. In patients with spondyloarthritis, platelets play a more central role in pathogenesis and are more likely to show an intimate relationship with disease activity than in patients with rheumatoid arthritis.

Kang et al [11] studied the correlation of platelet indices with inflammation markers and MRI findings in patients with axial spondyloarthritis. They found that platelet indices such as PDW and MPV may reflect the imaging changes on MRI. There was an inverse relationship between these indices and the disease activity. Inflammation suppressed the megakaryopoiesis, leading to decreased platelet size. Moreover, platelet consumption could have caused this observation. This relationship was even stronger in individuals with a shorter disease duration. As CRP levels and MRI findings are moderately correlated, biomarkers with better predictability are required. However, there were some drawbacks in the study as they included smokers, which could have altered the platelet indices, and they did not provide information on whether these findings were the same for both patients with AS and nr-axSpA.

Although there is ample literature focusing on platelet indices and their usability, reliability, availability, and low cost in the field of inflammatory rheumatological disorders, to date, no study has focused on their use in patients with nr-axSpA.

In this study, we will evaluate the viability of platelet indices in patients with nr-axSpA and determine whether they correlate with other objective evidence of inflammation, such as CRP levels and MRI sacroiliac joint findings, as well as clinical disease activity scores, such as BASDAI and ASDAS-CRP. Patients with nr-axSpA are expected to have a higher probability of having normal CRP levels and MRI findings. Therefore, this study is vital because this subgroup of patients has not been specifically studied previously, and there is a much greater need for alternative markers of disease activity in this subgroup.

### Axial Spondyloarthritis Spectrum, Pathophysiology, and Differences

According to the literature, at least 10% of patients with nr-axSpA progress to r-axSpA or AS, as observed during a follow-up of 2 years [19,20]. There is evidence suggesting r-axSpA as a continuation of nr-axSpA, as MRI changes, such as bone marrow edema, transform into structural changes such as erosions and sclerosis, which are detected on X-rays. Reports suggest that some of these patients with nr-axSpA have a self-limiting phenotype that never progresses and, therefore, should not be considered the same as AS [10]. The term AS was coined in 1904 by Eugene Frankel and was perceived as a disease causing inflammation in the sacroiliac joint, further progressing to the spine, and causing fusion of the axial skeleton [21].

According to studies in China, Europe, and the United States, a variable number of patients (1%-60%) may progress over the course of 2 to 15 years. Approximately 30% of patients with nr-axSpA may never progress to AS despite high disease activity, raised ESR, or CRP levels [21]. The CORRONA (Consortium of Rheumatology Researchers of North America) study showed a higher prevalence of nr-axSpA among women. The French and German studies showed the same pattern. The female patients had less structural damage and more peripheral symptoms [21]. Although patients with nr-axSpA had less structural damage, they showed an equivalent burden of the disease in terms of disease activity indices, such as the BASDAI, and functional indices, such as the Bath Ankylosing Spondylitis Functional Index, pain, quality of life index, and fatigue. Some studies showed that patients with nr-axSpA have higher disease activity and lower quality of life than patients with AS. Hip arthritis was more prevalent in patients with nr-axSpA, and these patients had future complications related to joint replacement surgeries. The CORRONA study also showed that impairment of activity was much greater in patients with nr-axSpA [21].

### Measuring Disease Activity

The main variables used are MRI findings and CRP levels. MRI can detect early inflammation in the sacroiliac joint compared to radiographic changes, which appear at later stages. Inflammatory lesions such as bone marrow edema are seen along the sacroiliac joint and vertebrae.

### Pitfalls in CRP and MRI

There is some disagreement regarding the use of CRP level as a marker of inflammation. Some studies have shown that CRP levels do not correlate well with disease activity. Some patients may have elevated CRP levels despite having low disease activity, and vice versa. Lower CRP levels are found in Asians and in patients with nr-axSpA. Nr-axSpA predominantly affects female individuals with lower CRP levels. CRP levels may be normal in about 40% to 60% of patients with axial spondyloarthritis, and they may be easily affected by BMI, treatment, and obesity. Studies have shown that a single CRP level may be insufficient to label a person as CRP-negative, and repeat CRP levels may be required in such cases, adding to the cost of managing the disease. Finally, inflammatory markers have shown poor predictability for early sacroiliitis [7,21-23].

Limitations of MRI findings include the variability in time between the onset of structural damage and clinical features. Moreover, bone marrow edema may be observed in otherwise healthy individuals, athletes, and pregnant women. Furthermore, a lack of training of both radiologists and rheumatologists in interpreting the MRI findings and determining a clinic-radiological correlation also contributes to the underdiagnosis or overdiagnosis of spondyloarthritis [22]. In a unique way, patients with nr-axSpA may show erosions without bone marrow edema, suggesting that early intervention might affect how the disease evolves [10].

MRI findings should also be interpreted in a clinical context. The lesions are not specific for axial spondyloarthritis, and they can be seen in various other conditions that are mechanical in nature or may be just findings in an otherwise normal individual. A total of 30% of patients with axial spondyloarthritis who are HLA B27 positive may not show bone marrow edema. Studies have shown that positive MRI may be visualized in only up to 15% of men who are HLA positive. Higher erosion is observed in patients with nr-axSpA than in those with AS, whereas this is reversed in fat metaplasia, suggesting that the latter might be the cause of bony structural changes [10].

Both MRI and CRP levels are insufficient to evaluate the disease activity status in patients with axial spondyloarthritis, especially in patients with nr-axSpA. Therefore, there is a need to search for alternative biomarkers that are reliable, inexpensive, and correlate well with clinical disease activity.

### Platelet Indices: Use as Inflammatory Markers

Platelet indices include various parameters related to platelets in the automated Coulter report of complete blood count. These different indices reveal various aspects of platelet morphology and kinetics. Different machines may provide different indices, with the key indices being PC, MPV, PDW, and plateletcrit. All these are readily accessible and require no extra cost from the patient. Many of these indices have been shown to be associated with inflammation in various inflammatory and infectious conditions [24].

Robinson et al [25] assessed RDW and MPV in patients with psoriatic arthritis and AS. The study showed significantly higher RDW and MPV in patients with active disease. A cutoff value of more than 12.4 for RDW had a sensitivity of 83% and specificity of 69%. In the case of MPV, a cutoff value greater than 8.4 had a sensitivity of 67% and specificity of 70%.

Byun et al [17] showed that PDW can be used as an alternate biomarker of active disease in cases of AS where conventional acute-phase reactants such as ESR and CRP are within normal range.

Kang et al [11] conducted a study on 161 patients with axial spondyloarthritis to correlate platelet indices with disease activity and MRI scores. They found that PC was positively correlated with sacroiliac bone marrow edema and disease activity. The platelet indices correlated well with the erosion score of sacroiliac joint MRI. However, the study also included smokers, which could be a confounding factor when measuring platelet indices.

All these previous studies had shortcomings in one way or another, as some did not include all platelet indices, some had confounding factors such as the inclusion of smokers, which themselves can alter the platelet indices, and some did not mention findings specifically for patients with nr-axSpA.

### Biomarkers

According to the World Health Organization, a biomarker provides information on diagnosis, prognosis, or disease activity and may be biological, chemical, or physical. Currently, CRP level is the sole biomarker used in spondyloarthritis. Although the treatment escalation is based on CRP elevation, it is a marker of generalized inflammation rather than being specific for spondyloarthritis. Various biomarkers have been studied for similar purposes in patients with spondyloarthritis. They can be categorized into different subtypes, such as markers for inflammation, bone homeostasis, antibody-associated, microbiome-associated, genetic markers, and miscellaneous. Along with the CRP level, the CRP to albumin ratio has also been proven to correlate with the disease activity score in axial spondyloarthritis. Other inflammatory markers studied were serum amyloid A; pentraxin 3; ratio of fibrinogen to albumin; adipokines such as leptin and omentin 1; cytokines such as TNF-α, IL-6, IL-17, IL-22, IL-31, and IL-33; and markers of bone homeostasis such as sclerostin, bone morphogenic proteins, matrix metalloproteases, osteoprotegerin, vimentin, and osteocalcin. Among the miscellaneous markers, complement 3 has been shown to correlate with disease activity and cardiometabolic risk. Despite all the biomarkers that have been studied and shown to correlate with disease activity, few are used in day-to-day practice. Accessibility, cost, and efficacy in predicting outcomes are major barriers to using these biomarkers in clinical practice [26]. A study showed that serum fetuin A levels were lower in patients with radiographic sacroiliitis and can serve as a marker for more severe disease. However, they showed no such correlation with spinal involvement [27]. Other markers of endothelial dysfunction, such as interleukin 18, endothelin 1, and soluble intercellular adhesion molecule 1, showed positive correlation with CRP and ESR in patients with AS and psoriatic arthritis [28]. Certain immunological biomarkers have also been studied to determine the phenotypes of axial spondyloarthritis and its activity levels, such as interferon gamma, interleukin 10, interleukin 17A, interleukin 22, interleukin 17f, 23 anti-CD74, zonulin, and monocyte chemoattractant protein 1 [29].

### Research Gap Analysis

Both AS and nr-axSpA show similar impact in terms of disease activity and functional deterioration [20]. Responsiveness to therapy depends on disease activity measured solely by CRP or MRI [7]. Research has been conducted to identify new markers of disease activity across various inflammatory diseases such as rheumatoid arthritis, scleroderma, AS, and systemic lupus erythematosus [13-18]. The research has shown that platelet indices are reliable and inexpensive markers of active disease. They are cheaper and as reliable as CRP for evaluating disease activity. Despite extensive literature showing that platelet indices can be used for measuring disease activity in numerous rheumatic conditions, no such literature is available for patients with nr-axSpA. The previous studies that tried to address this issue did not include all platelet indices in their analysis, and the final statistics did not show a separate analysis of the nr-axSpA group either. This group of patients invariably has lower levels of CRP; therefore, they may be more readily labeled as CRP-negative. Hence, it is imperative to establish new objective markers of inflammation that can be used on a day-to-day basis for quantifying the level of inflammation, especially in patients with nr-axSpA. This can lead to better management and prognosis, even in patients with clinical-serological desynchrony. This study would bridge the abovementioned gaps by evaluating patients with nr-axSpA and comparing them to patients with AS as well as controls, thereby showing stronger evidence of how platelet indices can differentiate between active and inactive disease. A comparison with other biomarkers and clinical disease activity indices can prove their application to be even better as stand-alone or composite markers of inflammation.

### Aim

This study aimed to evaluate whether platelet indices are reliable biomarkers for measuring disease activity in patients with nr-axSpA.

### Objectives

This study has the following aims:

1. To evaluate whether platelet indices can differentiate between active and inactive disease in patients with nr-axSpA.
2. To compare platelet indices with other proven measures of disease activity in axial spondyloarthritis, such as CRP levels and MRI findings.
3. To compare the systemic immune inflammation index (SII) with other clinical disease activity scores in patients with nr-axSpA.
4. To determine whether biomarkers such as the CRP to albumin ratio, complement 3, neutrophil to lymphocyte ratio, and platelet to lymphocyte ratio correlate with disease activity in patients with nr-axSpA.

## Methods

### Study Design and Duration

This cross-sectional, case-control study will be conducted from June 2024 to December 2027.

### Ethical Considerations

This study received ethical clearance from the institutional ethics committee (Datta Meghe Institute of Higher Education and Research) on January 6, 2024 (DMIHER[DU]/IEC/2023/59). The study participants will be provided with a consent form in their native language and English explaining the study in detail. Participants’ identification data will be kept confidential. The participants will have full autonomy to opt out of the study at any point.

### Inclusion Criteria

All patients diagnosed with axial spondyloarthritis as per the ASAS criteria and who provide consent will be included in this study. The cases will be divided into 2 groups. The first group will consist of patients with nr-axSpA (patients not presenting with radiographic sacroiliitis as per the modified New York Criteria), and the second group will consist of patients with radiographic sacroiliitis (either bilateral grade 2 sacroiliitis or unilateral grade 3 sacroiliitis). Normal healthy individuals will be included as controls.

### Exclusion Criteria

Patients with metabolic disorders; recent pregnancy; known cases of tuberculosis, brucellosis, or active infections; malignancy; trauma; crystal arthropathy; a history of vasculitis or hemoglobinopathies; smokers; patients on antiplatelet drugs; and those who do not consent to participate will be excluded.

### Controls

Age and sex matched, relatively healthy individuals with no known diseases, and no factors affecting the platelet indices and other acute-phase reactants will be included as a control group.

A detailed history will be obtained from all patients. Convenience sampling will be performed, and participants will be recruited consecutively from outpatient clinics. Subsequently, all included patients (as per ASAS classification criteria) will be sequentially divided into group A (nr-axSpA) and group B (AS or r-axSpA) based on radiographic sacroiliitis as per modified New York criteria. The control group will consist of healthy individuals. Patients will be clinically evaluated for disease activity status as per BASDAI, ASDAS-CRP, and SII scores. For BASDAI, a score of 4 or more will be considered as active disease. For ASDAS-CRP, a score of 1.3 or less will be considered as inactive disease, 1.3 to less than 2.1 as low activity, 2.1 to 3.5 as high disease activity, and a score of more than 3.5 as very high disease activity. For SII score, calculation will be done by formula (PC × neutrophil count or lymphocyte count) and will be correlated with other established clinical disease indices, as SII does not have a defined cutoff value for spondyloarthritis [30,31]. Patients’ blood samples will be collected for complete blood count (for platelet indices such as PC, plateletcrit, PDW, and MPV via Coulter reports), CRP via immunoturbidimetry, ESR (by Westergren’s method), HLA B27 by polymerase chain reaction, and complement 3 via turbidimetry. Neutrophil to lymphocyte ratio (cutoff value of 1.95 for high or very high disease activity) and platelet to lymphocyte ratio (cutoff value of 115.6 for active inflammation) will be calculated from the CBC Coulter report. CRP to albumin ratio (cutoff value of 0.75 for active inflammation) will also be included. None of the blood samples are fasting samples. Subsequently, patients will undergo pelvic MRI that will be read by 2 radiologists with a minimum of 10 years of experience in inflammatory pathologies. Both radiologists will independently look for radiological evidence of active inflammation. Spondyloarthritis Research Consortium of Canada (SPARCC) scoring will be used to grade the inflammation of the sacroiliac joint via MRI. This scoring will take active lesions from the sacroiliac joint using T2- sequences from MRI. Coronal sections will be used to identify these lesions. Each sacroiliac joint will be divided into 4 areas. A score of 0 will be assigned to areas with a normal signal intensity, and a score of 1 will be assigned to areas with an increased signal. The joints having an increased signal will receive an additional score of 1, and if the depth of the lesion is >1 cm, then another additional score of 1 per slice will be assigned. This scoring will be performed for 12 slides, and the maximum score could be 72 [32]. Both the pathologists and radiologists will be completely blinded to the included participants’ group. The MRI scoring will be done individually by 2 different radiologists. In the groups, the platelet indices (PC, plateletcrit, PDW, and MPV) will be compared with the levels of CRP, ESR, C3, neutrophil to lymphocyte ratio, platelet to lymphocyte ratio, CRP to albumin ratio, clinical disease activity scores (BASDAI, ASDAS-CRP, and SII), and with MRI-based SPARCC score.

### Sample Size

#### Sample Size With Justification

Sample size has been calculated with the help of Epi Info (version 7.2.2.2; EPI INFO), which is a trademarked tool from the Centers for Disease Control and Prevention.

Robinson et al [25] reported that the sensitivity of MPV values greater than 8.4 femtoliters in AS was 67%. Thus, in this study, *P*=0.67. The number of participants required for this study will be 196.53 ~ 197, with a power of 90%.

The formula used for the sample size calculation is as follows:




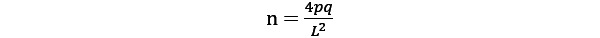




Where

n = required sample size; *P*=0.67, as per the study by Robinson et al [25]; q = 1 - p; and L = precision = 10%/

#### Calculation

Here, *P*=0.67, q = 1 – *P*=0.33, and loss% = 10%.




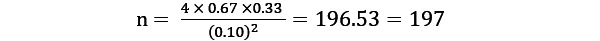




### Statistical Analysis

Statistical analysis will be performed with the help of Epi Info (version 7.2.2.2; EPI INFO), which is a trademark tool from the Centers for Disease Control and Prevention.

Descriptive statistical analysis will be performed to calculate the means with corresponding SDs. The test of proportion will be used to find the standard normal deviate (Z) to compare the difference in proportions. Chi-square test will be performed to determine the associations. The receiver operating characteristic curve will be used to find the area under the curve, followed by the Youden Method, which will use the cutoff value of different parameters for outcomes. These cutoff values will be used in multivariable logistic regression models to identify radiographic sacroiliitis and active disease. Sensitivity, specificity, positive predictive value, and negative predictive value will be calculated to determine the efficacy of the diagnostic tools. A *P* value of less than .05 will be considered to indicate statistical significance.

Categorical variables will be presented as numbers and percentages, and continuous variables will be presented as median and mean (SD). For assessing the correlation of platelet indices (PC, MPV, plateletcrit, and PDW), C3, CRP to albumin ratio, neutrophil to lymphocyte ratio, and platelet to lymphocyte ratio with other measures of disease activity, such as BASDAI, ASDAS-CRP, SII, and MRI sacroiliac joint SPARCC score, linear regression will be used (univariate and multivariate). Cohen κ statistic will be used to assess interobserver difference, or interrater agreement, by measuring the agreement between 2 radiologists.

## Results

After approval from the ethics and scientific committees, data collection was started on February 1, 2025. As of July 25, 2025, 7 participants have been included in groups A and B each and 5 individuals in the control group. We expect to complete the study by December 2027 and publish the results thereafter by January or February 2028. This is a nonfunded study.

## Discussion

### Anticipated Findings

Nr-axSpA is often difficult to evaluate in terms of confident assessment of disease activity. Most of these patients have clinical symptoms without any objective signs in our daily markers of inflammation. Such cases pose a challenge for clinicians in determining whether to increase or change medications. Therefore, there is an urgent need to establish new and credible markers that are easily available and have a more central relationship with sacroiliac joint inflammation. Platelet indices available in the literature fulfill these parameters but are not yet included in the standard of care. This study seeks to probe this issue and provide a better understanding of whether platelet indices are valuable in real clinical scenarios.

Tian et al [33] conducted a literature review to see if CRP levels and MRI findings correlated with patients with axial spondyloarthritis. Eighteen studies involving more than 1300 patients were included in this analysis. Although CRP levels showed a good correlation with spinal inflammation on MRI, no such correlation was observed between CRP levels and sacroiliac joint inflammation.

Landewe et al [7] evaluated the possibility of patients with axial spondyloarthritis with an initial normal CRP being deemed CRP-negative, and subsequently presenting with a raised CRP the second time. A total of 106 patients were enrolled, and 25% of them had normal baseline CRP levels; 50% of these patients later showed elevated CRP levels (up to 16 weeks). The study emphasized the reevaluation of CRP levels after at least 4 weeks to confirm that the patient had normal CRP levels.

Rusman et al [34] evaluated the presence of MRI-based inflammation in patients with nr-axSpA who had high disease activity. SPARCC scoring was used to grade the inflammation on MRI of the sacroiliac joints and spine. A total of 69 patients were included in this study, and only 39% of them showed signs of active inflammation on MRI despite having high disease activity. Among patients who did not have any MRI lesions at baseline, 93% showed no signs even after 6 months, even though they had disease activity.

Magrey et al [21] elucidated that nr-axSpA represents a clear-cut phenotype with a disease burden similar to AS, and there is a lack of understanding of the severity of this phenotype even among rheumatologists. The deferral in initiating adequate treatment in such patients often leads to a decreased quality of life and an unwarranted delay in proper care. This study highlights the need to identify biomarkers to detect this phenotype early and to predict clinical responses to overcome these obstacles.

Byun et al [17] showed that CRP and ESR, although widely used as markers of inflammation in patients with AS, were not completely reliable due to false-negative and false-positive values. Platelet indices, such as PDW and PC, showed a good correlation with objective clinical disease activity scores such as BASDAI and ASDAS, and CRP. The study concluded that PDW is a suitable adjunctive marker of inflammation in patients with normal CRP levels.

Qian et al [35] evaluated patients of AS who were followed up for 6 months on anti-TNF therapy. These patients had higher PCs at the start of therapy than the controls, and they showed lower PCs on follow-up starting from the first month. Patients who did not respond to therapy had more PCs than those who did. PCs also correlated well with ESR and clinical disease activity scores.

### Conclusions

This study is expected to provide a decisive answer to whether platelet indices can be used as reliable markers of active disease in patients with nr-axSpA, especially in those with conventional inflammatory markers within the normal range and normal MRI findings of the sacroiliac joints.

## Abbreviations

AS: ankylosing spondylitis
ASAS: Assessment of Spondyloarthritis International Society
ASDAS: Ankylosing Spondylitis Disease Activity Score
BASDAI: Bath Ankylosing Spondylitis Disease Activity Index
CORRONA: Consortium of Rheumatology Researchers of North America
CRP: C-reactive protein
ESR: erythrocyte sedimentation rate
MPV: mean platelet volume
MRI: magnetic resonance imaging
nr-axSpA: nonradiographic axial spondyloarthritis
PC: platelet count
PDW: platelet distribution width
r-axSpA: radiographic axial spondyloarthritis
RDW: red cell distribution width
SII: systemic immune inflammation index
SPARCC: Spondyloarthritis Research Consortium of Canada
TNF: tumor necrosis factor

## Data Availability

The datasets generated or analyzed during this study are available from the corresponding author on reasonable request.
